# Reproductive health communication between mother and adolescent daughter in Bangladesh: a cross-sectional study

**DOI:** 10.1186/s12978-019-0778-6

**Published:** 2019-07-24

**Authors:** Muhammad Zakaria, Junfang Xu, Farzana Karim, Feng Cheng

**Affiliations:** 1 Department of Communication and Journalism, University of Chittagong , Chittagong 4331, Bangladesh; 2 Center for Health Policy Studies, School of Public Health, Zhejiang University School of Medicine, Hangzhou, 310058 China; 3 Research Center for Public Health, School of Medicine, Tsinghua University, Beijing, 100084 China

**Keywords:** Communication, Reproductive health, High school girls, Mothers, Bangladesh

## Abstract

**Background:**

Parent-adolescent reproductive health (RH) communication is one of the potential sources of information for adolescents on the topic. Given that female adolescents in Bangladesh are faced with increasing RH-related risks, it is important to understand how parents communicate about RH to their adolescents from the adolescents’ perspectives. Therefore, the aim of this study is to explore the status of mother-adolescent daughter communication on reproductive health in Bangladesh.

**Methods:**

A cross-sectional study targeting female students was conducted in five high schools in Chittagong based on a self-administered questionnaire survey. A description method was used to describe the characteristics of mother-adolescent daughters’ communication on RH including the frequency, type and the quantity of topics. Bivariate and multivariate logistic regression analyses were performed to explore the factors influencing mother-adolescent daughter communication.

**Results:**

In the study, 1174 female students aged from 13 to 19 years old were included. The main source of knowledge on RH was from their mother (62%), and the mother was the person who communicated first on RH with adolescent students. The topics of mother-daughter communication were mainly focused on menstruation issues (> 80%). Multivariate logistic regressions showed that Hindu students, students with good RH knowledge, adolescents’ mothers having good RH knowledge, mothers with high media use, good mother-daughter relationship, daughters’ regular general communication with mothers, and students’ perceiving comfort in RH communication with their mothers were reported as significant predictors for a good RH communication status. On the contrary, students having family members numbering more than four, whose primary source of reproductive health information was friends/classmates as well as media were less likely to have better RH communication with mothers.

**Conclusions:**

The overall communication on reproductive health between adolescent daughters and their mothers was not good. This study suggests for conducting qualitative research investigating the socio-cultural context within which the RH communications happen. and how to address the obstacles that might hinder this communication.

## Plain English summary

Given that female adolescents in Bangladesh are faced with increasing reproductive health (RH)-related risks, we aim to understand how parents communicate about RH to their adolescents from the adolescents’ perspectives.

A cross-sectional study targeting female students was conducted in five high schools in Chittagong based on a self-administered questionnaire survey.

One thousand one hundred seventy-four female students aged from 13 to 19 years old were included. The main source of knowledge on RH was from their mother (62%).Mother-daughter communication was  mainly focused on menstruation issues (> 80%). Respondents both with some and good RH knowledge, Hindu students, adolescents’ mothers having good RH knowledge as well as high media use, mother-daughter having friendly relationship, also regular general communication between them, and students’ who perceived comfort in RH communication with their mothers had a higher likelihood to have a good RH communication status. However, students having family members numbering more than four, whose primary source of RH information was friends/classmates as well as media were less likely to have better communication with mothers regarding RH.

This study suggests for conducting qualitative research investigating the socio-cultural context within which the RH communications happen.

## Background

Improving the sexual and reproductive health (SRH) of adolescents is one of the primary concerns of Sustainable Development Goals (SDGs) [1]. Evidence has shown that mothers are the preferred source of sexual and reproductive health knowledge, information and discussion for adolescent girls worldwide [2–7]. However, most mothers are reluctant to talk with their children about sexual issues in some countries, including Bangladesh, because of the influence of traditional culture as well as religious dogma, which causes them to tend to limit the discussion to safe topics [8]. In addition, adolescents, especially girls, are often unwilling to look for information on SRH under an orthodox society with cultural and religious impediments [4]. Therefore, adolescent daughters are affected by a lack of communication, information and education on SRH, which causes teenage girls, especially those who live in rural areas, to be more prone to high risk behavior and adverse outcomes for sexual health.

Bangladesh ranks amongst the highest in the proportion of child marriages as well as early childbearing globally [9]. The adolescent fertility rate in Bangladesh also follows closely with that in Asian countries and some African countries [10]. In Bangladesh, social norms and cultural traditions encourage girls to marry at an early age. Therefore, a large proportion of marriages still take place before the legal age of 18 years old, and married teenage girls have to prove their fertility soon after marriage [11]. The 2014 Bangladesh Demographic and Health Survey (BDHS) showed that 59% were married by the age of 18 years [12]. The survey also revealed that 31% of married adolescents with the age of 15–19 years old in the country are already mothers or pregnant with their first child while nearly 70% give birth by the age of 20 [12]. Pregnancy related maternal mortality rate among the married youngest girls (15–19 years) is higher in 2016 (144 deaths per 100,000 live births) than that in 2010 (75 deaths per 100,000 live births) [13, 14]. These adolescent mothers have a higher likelihood of experiencing complications during pregnancy and a higher probability of maternal death. Furthermore, children born to very young mothers are at greater risk of illness and death [15]. The maternal mortality rate for adolescents is almost twice the national figure [15]. In addition, neonatal mortality, infant mortality and under-5 mortality rates are also higher among females aged < 20 years compared to those in the low-risk age group of 20–39 years [13]. Therefore, better communication between mother-daughter may help to improve the reproductive health knowledge, attitudes and practices of adolescent girls.

Studies on parent-adolescent reproductive health communication have been conducted in many high income countries [16–18]. However, no study has investigated the mother-daughter reproductive health communication in Bangladesh. Therefore, the aim of this study is to analyze adolescent girls’ communication on SRH issues with their mothers by investigating the frequency of mother-adolescent daughter communication regarding reproductive health, type of interaction and the topics covered during dyadic communication; and to explore the factors associated with mother-daughter communication on reproductive health issues.

## Methods

### Study design and settings

This study was cross-sectional research using a self-administered questionnaire survey (the questionnaire was examined with a high reliability and validity) from February 11 to February 24, 2018. Study settings were five high schools located in the Chittagong district of Bangladesh including three schools in rural areas and two schools in urban areas. Chittagong covers the south-eastern part of the country, with a total area of 33,771.18 km^2^ and a population of 28,423,019 in 2011 [19].

For the selection of schools, both purposive and random sampling methods were used. For urban participants, Chittagong City was selected purposively since it is the only metropolitan city of the district with a diversified population. Then one city corporation run (autonomous) high school and one private high school respectively named ‘Krishnakumari City Corporation School’ and ‘Ispahani Public School & College’ were randomly selected for the study from the lists of above mentioned types of schools. Government high schools were not included as the classes of all government schools were suspended due to the public examinations during the study period. For collecting data from rural respondents, two Upazilas, Raozan and Satkania, the former from the north part and the latter from the south part of the Chittagong District were selected by lottery method. Then two high schools, named ‘Satkania Girl’s High School’ and ‘Urkirchar High School’ from each of the two Upazilas were finalized randomly as the study setting is using simple random sampling technique. Rangamati District was taken as it is the largest district in Chittagong Hill Tracts (CHT) area and a high school named ‘Vedvede Pouro High School’ was selected by following random sampling technique. We selected CHT area to include the Ethnic and Non-Muslim groups of respondents in the study. In this way, five schools were selected for the institution-based cross-sectional survey.

### Study population and sample size

The study population was female students aged 13–19 years attending grade 9 and grade 10 of Chittagong schools. The sample size (*n* = 1067) was determined considering the following assumption: *p* = 50%, significance level 5% (α = 0.05), $\mathrm{Z}\frac{\alpha }{2}=1.96$, margin of error 3% (d = 0.03). Assuming a 10% nonresponse rate, the final sample size was: n = 1067 + 107 = 1174.

In recruitment, the inclusion criteria were the adolescent girls who were living with their mothers in the family and who had started menstruation at least 1 year preceding the survey. The girls whose mothers were not alive and those who were not willing to participate in the study were excluded. Finally, 1174 female students were included in the study. The mean age of the respondents was 14.93 years (SD = .87). Of them, 630 (53.7%) were attending grade 10 while 544 (46.3%) were the students of grade 9 at the high schools.

### Measure of outcome variables

The main outcome variable “mother-daughter communication on adolescent health” was measured using three variables: 1. frequency of communication between mother and daughter on adolescent issues (‘high’- at least once in 1 month and ‘low’- once in more than 1 month); 2. quantity of topics covered in discussion (‘more’- at least five topics or ‘few’- less than five topics); 3. type of interaction (‘mutually interactive’ or ‘one-sided’). One-sided implies either ‘respondents she (daughter) mostly communicates’ or ‘mother mostly communicates’; and ‘mutually interactive’ contains ‘both mother and respondents communicate equally.’ Furthermore, for measuring the quantity of topics covered in the discussion 12 topics relating to reproductive health issues including biological aspect, prevention aspect, safe and risky behaviour were listed. The respondents were asked to tick the topics they had covered during a discussion with their mother. The responses for each topic were dichotomized as ‘Yes’ (1) or ‘No’ (0). The quantity ranging from 0 to 12 was dichotomized using the mean (which was 4.46) as a cut-off value so that the quantity above the mean value was defined as ‘more’ (at least five) topics and below the mean value was defined as ‘few’ (less than five) topics covered during discussion between mother and adolescent daughter [20].

Finally, after getting dichotomous scores of three dependent variables, the scores of these variables were added together to generate the main outcome variable which was the overall status of mother-adolescent daughter reproductive health communication. The composite score was dichotomized using the mean (which was 1.80) as a cut-off value, so that a score above the mean value was coded as ‘good communication status’, and a score below the mean value was defined as ‘poor communication status’ [20].

### Statistical analysis

A description method was used to describe the characteristics of mother–adolescent daughters’ RH communication including the frequency, communication type and quantity of topics. Bivariate and multivariate logistic regressions were performed to explore the factors influencing mother-adolescent daughter communication. SPSS 16.0 software was used for the statistical analyses with an alpha level of 0.05.

## Results

### Socio-demographic characteristics

Table 1 shows the socio-demographic characteristics and household information of the participants. A total of 1174 adolescent girls were incorporated in the study. More than half of the students (52.9%) were from rural areas, and 47.1% of them were from urban areas. The majority of the respondents (71.6%) were Muslim followed by Buddhist and Hindu accounting for 216 (18.4%) and 108 (9.2%) respectively. Regarding the family size, 819 (69.8%) were more than four in number. 375 (31.9%) students reported their mother completed a secondary level of education followed by higher secondary (20.4%), primary (15.8%), bachelor (12.4%), masters (10.9%) and illiterate (8.5%). The majority of students’ mothers (1057, 90%) were housewives. Regarding estimated average monthly income of the household 25.2% respondents reported their parents’ monthly income to be up to BDT (Bangladesh Taka) 10000(118.9 US$), whereas 27.7% reported it to be BDT > 10000–25000(118.9 US$-297.2 US$), 27.5% to be BDT > 25000–50000(297.2 US$-594.4 US$) and 19.6% to be BDT > 50000(594.4 US$).

**Table 1:** Socio-demographic characteristics of high schools’ female students and parents. (*N* = 1174)

Variable	Frequency	Percentage (%)
Section		
Arts	319	27.2
Commerce	425	36.2
Science	430	36.6
Area of residency		
Rural	621	52.9
Urban	553	47.1
Religion		
Muslim	841	71.6
Hindu	108	9.2
Buddhist	216	18.4
Christian and others	9	0.8
Race/ethnicity		
Bengali	975	83.0
Ethnic	199	17.0
Family size (persons)		
3–4	355	30.2
5 and above	819	69.8
Educational status of the father		
Illiterate	69	5.9
Primary	184	15.7
Secondary	314	26.7
Higher Secondary	209	17.8
Honors/Degree	171	14.6
Masters	227	19.3
Educational status of the mother		
Illiterate	100	8.5
Primary	186	15.8
Secondary	375	31.9
Higher Secondary	239	20.4
Honors/Degree	146	12.4
Masters	128	10.9
Occupation of father		
Agriculture	132	11.2
Expatriate	99	8.4
Business	468	39.9
Service	304	25.9
Others	171	14.6
Occupation of mother		
Housewife	1057	90.0
Business	13	1.1
Service Holder	36	3.1
Teacher	49	4.2
Others	19	1.6
Household income (BDT)		
Up to 10,000	267	25.2
>10,000-25,000	294	27.7
>25,000-50,000	291	27.5
>50,000	208	19.6

### RH knowledge, menstrual hygiene, and media use

Table 2 showed the knowledge, attitude on RH and use of social media. More than half of the respondents (54.5%) had the perception of some extent knowledge on adolescence issues while more 321 (27.3%) reported having their good knowledge pertaining to this topic. In addition, 213 (18%) respondents knew little about RH. According to the reports of the respondent’s perception, 437 (37.2%) mothers had some extent knowledge on RH matters whereas 420 (35.8%) mothers had good knowledge. Furthermore, 188 (16%) respondents reported that their mothers knew a little about reproductive while 129 (11%) were not sure about the level of knowledge of their mothers. Of respondents, two third (67.8%) opined that they had a positive attitude towards discussion RH issues elaborately with mother, elder sister, friends, and relatives, while 378 (32%) had a negative attitude about open discussion regarding this topic. Regarding the menstrual hygiene management, 367 (31.3%) used cloth during their menstruation cycle while 807 (68.7%) used sanitary pad during period. The result shows that 755 (55.8%) acknowledged their high use of mass media, while more than half of respondents reported that their mothers had low access to mass media amounting 607 (51.7%). In addition, of respondents, 311 (26.5%) reported their access to social media, whereas 205 (17.5%) acknowledged their mothers’ use of social media.

**Table 2:** Knowledge, attitude and use of different media (*N* = 1174)

Variables	Frequency	Percentage
Respondents’ perception of RH knowledge		
Poor knowledge	213	18.1
Some extent knowledge	640	54.5
Good knowledge	321	27.3
Respondents’ perception of mothers RH knowledge		
Not sure	129	11.0
Poor knowledge	188	16.0
Some extent knowledge	437	37.2
Good knowledge	420	35.8
Respondents’ attitude towards RH discussion		
Negative	378	32.2
Positive	796	67.8
Menstrual hygiene management		
Use cloth during menstruation	367	31.3
Use sanitary pad during period	807	68.7
Level of respondents’ mass media use		
Low	519	44.2
High	655	55.8
Level of mothers’ mass media use		
Low	607	51.7
High	567	48.3
Respondents’ social media use		
No	863	73.5
Yes	311	26.5
Mothers’ social media use		
No	965	82.5
Yes	205	17.5

*Note. RH* Reproductive Health

### The source of knowledge on RH

**Fig. 1:**
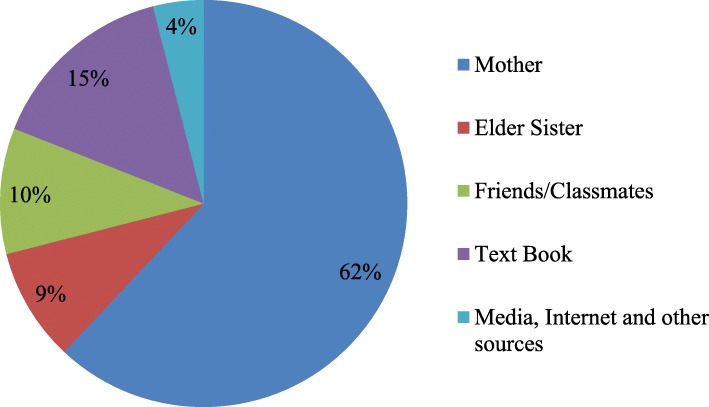
The source of knowledge on sexual and reproductive health for adolescents in Bangladesh

**Fig. 2:**
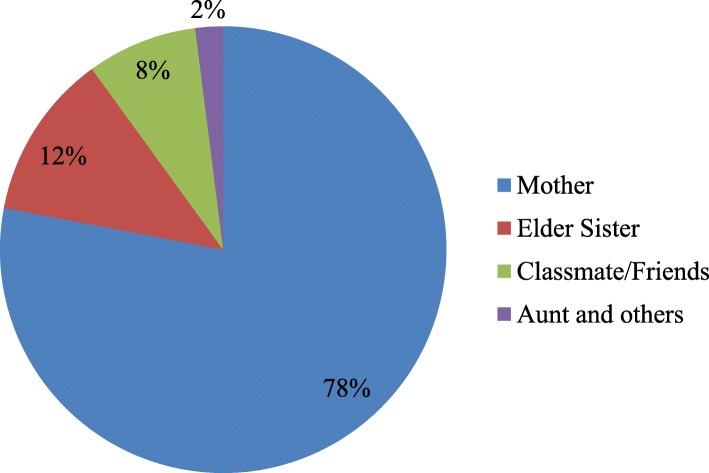
The person with whom students communicated first on reproductive health issues in Bangladesh

Figures 1 and 2 display the prime source of knowledge regarding reproductive health and the person with whom the respondents communicated first. Most of the adolescent girls (62%) acknowledged their mothers as the primary source of knowledge on reproductive health issues, followed by textbook (15%), classmates and friends (10%) and elder sister (9%). Moreover, more than three-fourths of the girls reported mothers as the person with whom they had the first discussion about sexual and reproductive health followed by elder sister (12%), classmates or friends (8%) and aunt and others (2%).

### Topics covered in mother-daughter RH discussion

**Fig. 3:**
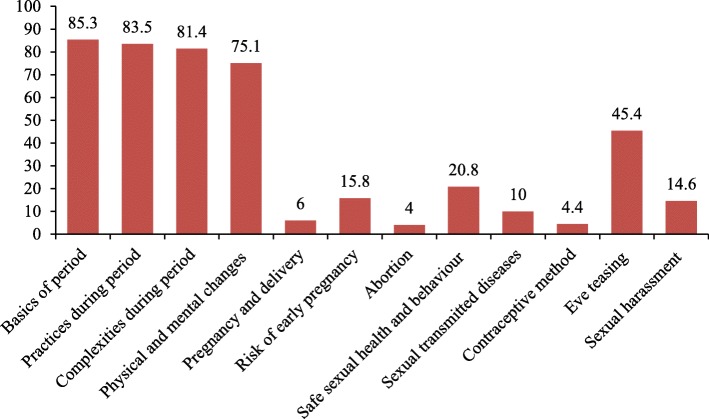
Topics covered in mother-daughter communication on sexual and reproductive health in Bangladesh

Figure 3 demonstrates the topics covered in communication on reproductive health between mother and adolescent. The majority of respondents’ discussion with their mother was limited to four topics relating to period and changes during puberty. The topics covered in the interpersonal communication were: basics of period (85.3%), safe practices during menstruation (83.5%), complexities during period (81.4%), physical and mental changes during puberty (75.1%), Eve teasing (45.4%), safe sexual health and behavior (20.8%), risk of early pregnancy (15.8%), sexual harassment (14.6%), sexually transmitted diseases (10%), pregnancy and delivery process (6%), contraceptive methods (4.4%) and abortion (4%).

### Mother-daughter communication on RH

More than one-third (34.4%) of students had poor communication on reproductive health with their mother (Table 3). 812 (69.2%) reported that they had a high frequency (at least once in 1 month) of communication with their mother whereas 362 (30.8%) had a low frequency (once in more than 1 month). Moreover, 753 (64.1%) students had mutually interactive communication with their mother, and 421 (35.9%) with one-sided communication. In respect to the quantity of topics, 618 (52.6%) respondents mentioned some content (maximum 4 out of 12) had been covered in the communication with their mother, while less than half (47.4%) of the respondents acknowledged they discussed more topics (more than 4 out of 12) related to reproductive health. As to initiating communication between mother and daughter, 798 (68.5%) respondents reported that their mother commenced interaction with them about period and reproductive issues, while about one third (31.5%) of the respondents reported of taking initiative on their own for interpersonal communication on RH with their mothers. Regarding the starting time of RH communication, more than two-thirds (67.2%) of the respondents reported that mother-daughter communication on adolescence health started after menarche, while the rest (32.8%) reported commencing this type of intra-family interaction before experiencing menarche.

**Table 3:** Mother-daughter communication status on RH in Bangladesh

Communication	Frequency	Percentage
The overall status of mother-daughter communication on RH		
Good	770	65.6
Poor	404	34.4
Frequency of communication		
High (Once in one month)	812	69.2
Low (Once in more than one month)	362	30.8
Communication type		
Mutually interactive	753	64.1
One-sided	421	35.9
Topics covered during communication		
More	556	47.4
Few	618	52.6
The initiator of mother-daughter communication on RH		
Daughter	367	31.5
Mother	998	68.5
Starting time of RH communication		
After menarche	761	64.8
Before menarche	413	35.2

*RH* Reproductive Health

### Factors influencing RH communication between mother and daughter

The result of multiple logistic regression models revealed that most of the variables related to knowledge, media use, relationship and attitude towards open discussion on RH had been found as a statistically significant predictor for having a good communication status between mother and daughter on adolescent health (Table 4). Moreover, Hindu students were 1.76 times more likely to have better communication than Muslim students (AOR = 1.76, 95% CI: 1.03–3.00). Students who had family members numbering more than four were less likely to have good discussion quality than their counterparts having family members numbering less than four (AOR = 0.69, 95% CI: 0.50–0.95).

**Table 4:** Odds of socio-demographic and other background characteristics predicting mother-adolescent communication on reproductive health

Variables (*N* = 1174)	Mother-daughter RH communication status			
	Good	Poor	COR (95% CI)	AOR (95% CI)
Religion				
Muslim	556 (66.1)	285 (33.9)	1.00	1.00
Hindu	84 (77.8)	24 (22.2)	1.79 (1.11–2.89)*	1.76 (1.03–3.00)*
Buddhist	127 (58.8)	89 (41.2)	0.73 (0.54–0.99)*	1.03 (0.43–2.50)
Christian and others	3 (33.3)	6 (66.7)	0.26 (0.06–1.03)	0.59 (0.11–2.97)
Race/ethnicity				
Ethnic	113 (56.8)	86 (43.2)	1.00	1.00
Bengali	657 (67.4)	318 (32.6)	1.57 (1.15–2.15)**	1.08 (0.42–2.81)
Area of residence				
Rural	263 (42.4)	358 (57.6)	1.00	
Urban	336 (60.8)	217 (39.2)	2.11 (1.67–2.66)***	
Family size (persons)				
3–4	253 (71.3)	102 (28.7)	1.00	1.00
5 and above	517 (63.1)	302 (36.9)	0.69 (0.53–0.90)**	0.69 (0.50–0.95)*
Educational status of the mother				
Illiterate	52 (52.0)	48 (48.0)	1.00	1.00
Primary	118 (63.4)	68 (36.6)	1.60 (0.98–2.62)	1.25 (0.71–2.20)
Secondary	258 (68.8)	117 (31.2)	2.04 (1.30–3.19)**	1.22 (0.70–2.15)
Higher Secondary	162 (67.8)	77 (32.2)	1.94 (1.20–3.13)**	1.01 (0.56–1.81)
Honors/Degree	103 (70.5)	43 (29.5)	2.21 (1.30–3.76)**	0.99 (0.51–1.95)
Masters	77 (60.2)	51 (39.8)	1.39 (0.82–2.36)	0.53 (0.26–1.7)
Respondents RH knowledge				
Poor	106 (49.8)	107 (50.2)	1.00	1.00
Some extent	426 (66.6)	214 (33.4)	2.01 (1.47–2.75)***	1.63 (1.15–2.30)**
Good	238 (74.1)	83 (25.9)	2.89 (2.01–4.18)***	2.01 (1.31–3.09)**
Perception of Mothers’ RH knowledge				
Poor	100 (53.2)	88 (46.8)	1.00	1.00
Not sure	58 (45.0)	71 (55.0)	0.72 (0.46–1.13)	0.86 (0.52–1.44)
Some extent	291 (66.6)	146 (33.4)	1.75 (1.24–2.49)**	1.32 (0.88–1.98)
Good	321 (76.4)	99 (23.6)	2.85 (1.98–4.11)***	1.93 (1.25–2.97)**
Primary source of RH knowledge				
Mother	509 (70.0)	218 (30.0)	1.00	1.00
Elder sister	61 (59.8)	41 (40.2)	0.64 (0.42–0.98)*	1.04 (0.64–1.67)
Friends/Classmates	57 (49.6)	58 (50.4)	0.42 (0.28–0.63)***	0.57 (0.37–0.90)*
Textbook	123 (67.2)	60 (32.8)	0.88 (0.62–1.24)	1.02 (0.69–1.49)
Media and others	20 (42.6)	27 (57.4)	0.32 (0.17–0.58)***	0.40 (0.20–0.78)**
Media use of mothers				
Low	364 (60.0)	243 (40.0)	1.00	1.00
High	406 (71.6)	161 (28.4)	1.68 (1.32–2.15)***	1.58 (1.19–2.10)**
Mother-daughter good relationship				
No	244 (55.1)	199 (44.9)	1.00	1.00
Yes	526 (72.0)	205 (28.0)	2.09 (1.63–2.68)***	1.36 (1.02–1.81)*
Mother-daughter general communication				
Irregular	97 (41.8)	135 (58.2)	1.00	1.00
Regular	673 (71.4)	269 (28.6)	3.48 (2.59–4.68)***	2.62 (1.87–3.66)***
Respondent’s attitude about a discussion on RH				
Negative	225 (59.5)	153 (40.5)	1.00	
Positive	545 (68.5)	251 (31.5)	1.48 (1.14–1.90)**	
Comfort of RH communication				
Uncomfortable	201 (55.4)	162 (44.6)	1.00	1.00
Comfortable	569 (70.2)	242 (29.8)	1.89 (1.47–2.45)***	1.45 (1.09–1.93)*

* *p* < .05; ** *p* < .01; *** *p* < .001; 1.0 = consonant; *AOR* Adjusted odds ratio, *COR* Crude odds ratio, *CI* Confidence interval, *RH* Reproductive health

Table 3 also demonstrates that the higher the level of knowledge the students and their mothers had on reproductive health, the better communication was had on RH. Moreover, the students whose primary source of reproductive health was friends/classmates, as well as the media, were less likely to have better communication with mothers (AOR = 0.57, 95% CI: 0.37–0.90 Vs. AOR = 0.40, 95% CI: 0.20–0.78) than those who got RH knowledge from their mother. Respondents’ mothers who had a high level of use of newspapers and TV had better communication than those who had a low media use (AOR = 1.58, 95% CI: 1.19–2.10). Those having a good mother-daughter relationship (AOR = 1.36), regular communication (AOR = 2.62), and comfortable communication (AOR = 1.45) tended to have good communication on RH. The odds of better mother-daughter communication were 1.36 times higher among the respondents who reported that there was a good relationship between mother and daughter than those who had no friendly relationship (AOR = 1.02, 95% CI: 1.02–1.81). In the same way, of respondents who had regular general communication with their mother had a greater association with their effective reproductive health discussion with odds of 2.62 compared with those who communicated irregularly (AOR = 2.62, 95% CI: 1.87–3.66). Furthermore, the respondents’ perceived comfort of communication on adolescent issues with their mother found a significant relationship with better mother-daughter communication regarding reproductive matters than their counterparts who felt discomfort during a discussion on reproductive health issues with their mothers (AOR = 1.45, 95% CI:1.09–1.93).

## Discussion

The frequencies of mother-daughter communication in this study is higher than that of other countries, for example, India, Ethiopia and Zimbabwe [5, 21–23] and lower than the USA [24]. The main reason for this high percentage of frequency could be due to the differences in culture. Traditionally, daughters in Bangladesh have a trusting relationship with their mothers due to gender homogeneity. They spend more time in the home with their mothers because movement outside of the house is restricted in rural areas due to its patriarchal culture. Moreover, in a girl’s adolescent period they usually do not have any credible access to information beyond the family, and they are not allowed to go outside except to school without parents’ permission. This explanation is also supported by the other findings of this study where mothers were considered the primary source of reproductive health knowledge for 62% of students, while 78% of students reported that their mother was the first person with whom they communicated after getting their first period. These were consistent with other studies in developing countries [21, 22, 25, 26].

The topics of mother-daughter communication were also explored in this study, which indicated the range of discussion was narrow and restricted to period and related topics. Studies reported that adolescents tended to discuss menstruation with their mothers [25]. The percentage of period-related topics as the content of communication was much higher than others [23–27]. Likewise, physical change as a topic was covered in 75% of communication which is lower than East Wollenga [22], but higher than other studies [26–28]. The difference in discussion rate regarding menstruation and physical change may be due to the differences in sources of information. In Bangladesh, these essential topics about puberty are usually discussed by mothers as they are the primary source of information for their daughters whereas, in different countries, adolescent girls are being informed on these issues by the media and school [29]. The discussion rate of other topics regarding reproductive and sexual health, assessed in this study, is much lower than other studies, such as, pregnancy and delivery process [16, 28], risk of early pregnancy/unwanted pregnancy [21, 22, 26], abortion [22], safe sexual health and behavior [26], sexually transmitted diseases [16, 30, 31], and contraceptive methods [28, 31]. These differences may be attributed to cultural and attitudinal incongruity. In Bangladesh, mothers usually prefer to limit their discussion to safe topics and are often reluctant to talk about sexual health despite the urgent need of it. Sometimes, mothers think that prior knowledge about sexual health may lead adolescents to become sexually active.

In our study, more than two-third of the respondents reported that mothers initiated RH discussion with them, which is consistent with other studies [8, 32]. It is also noticeable that among the respondents who started a discussion with mothers, 80% had first communication with mother after getting the menarche, which may imply that experiencing with this biological changes lead them to communicate with mothers. Another important thing is among the mothers who started communication with daughters, only 42% of them initiated to talk about this issue before first menstruation. In other words, most of the mothers didn’t start puberty discussion in advance. It may be due to the perception of taboo, conservative attitude and the traditional belief that being informed about sexual and reproductive issues in teenage a girl may engage in the risky sexual practice. Therefore, lingering to SRH communication may cause by these socio-cultural impediments.

This study showed that Muslim students were less likely to have better reproductive communication with mothers than those of Hindu students. But Bangladesh is one of the countries where the majority of the population (89%) is Muslim [33]. Religious spirit and beliefs among Muslim people discourage open discussion on sexual health issues, which may hinder mother-daughter communication. As observed, the students having a family size of more than four were less likely to have experienced good reproductive health communication with their mothers. Probably, students with larger families have an elder sister with whom the adolescent prefers to discuss puberty issues rather than with their mothers. This study’s findings also support this statement showing that 91.4% of respondents with family members of more than four have an elder sister. Moreover, the elder sister was the person with whom 12% of students communicated first regarding sexual health. Usually, girls who have an elder sister in the family are more likely to talk over different issues including adolescent health with the elder sister than the mother due to the smaller age gap between them.

Evidence has shown that knowledge is an essential determinant of better health status as gaining knowledge and being aware is the first step to adopting as well as sustaining recommended and safe health-related behavior and practice [34]. A knowledgeable girl and mother can comprehend the importance of reproductive health communication and form a favorable attitude to interact regarding this without reticence. Our study found respondent’s perception of her own knowledge on puberty is largely correlated with the presence of good communication quality. It may be attributable to students who have some information and who then might be interested in getting more knowledge through intra-family discussion, and the awareness they already have may direct the way for initiation of interaction. This finding is in agreement with other studies [21, 26, 28]. However, it is noticeable here that the present study assessed the students’ own perception about their level of knowledge as well their mothers’ knowledge which may not reflect their actual reproductive health knowledge.

In this study, the primary source of reproductive health knowledge was reported as the factor associated with a good communication level which showed that the students whose primary sources were friends/classmates, as well as the media, had a lower likelihood of having a good communication experience with their mother. This is due to the fact that a girl who obtained sexual health information from a classmate/friend may prefer to discuss this issue with them instead of their mother. Besides, homogeneity in some attributes between them such as age, sex, education and family status leads them toward further communication on this issue. In addition to this, the students who got reproductive knowledge from the media were less likely to communicate with their mothers. This may be their reliance on the media for health-related information resulting in the decline of the frequency of discussion with their mother. However, mothers’ media use appeared as one of the factors for having good communication quality between mother and daughter. An amicable relationship with their mother pushes the daughter towards self-disclosure to the mother and to discuss any issues openly. This study’s findings are similar to others [28] that show a strong association between amity in the mother-daughter relationship and their interaction regarding puberty. This study’s findings also support others demonstrating that regular general mother-daughter communication has a profoundly positive outcome on puberty related communication [35]. This study, furthermore, depicts that students’ perception of comfort of communication with their mother was reported as a significant determinant of good reproductive health communication. Thus, this study agrees with others [28].

### Limitations of the study

The study has some limitations. First, only mother-daughter communication on reproductive health from the perspective of daughters was reported on. Therefore, studies from the perspective of both mother and daughter would provide a more comprehensive picture. Moreover, question might be raised whether geographic and cultural variety in Bangladesh could challenge the claim of this study; the entirety of adolescent students, although respondents from different socio-demographic background were included as the study samples.

## Conclusion

In conclusion, mothers were reported as the primary source of knowledge and information about reproductive health issues for most of the adolescent girls. However, the communication included a narrow range of reproductive and sexual health issues and mainly covered menstruation-related topics, which may not be addressed to make teenage girls more capable with basic concepts.

This study suggests providing comprehensive RH education and behavioral change communication program in schools is important in Bangladesh. Moreover, studies conducting from the mothers’ perspective to have a comprehensive depiction and to identify predictors for the effective communication of sexual and reproductive health issues between daughters and mothers are urgent. Furthermore, conducting qualitative research investigating the socio-cultural context within which the SRH communications happen and how to address the obstacles that might hinder this communication may help a lot.

## Data Availability

All of the main data has been included in the results. Additional materials with details may be obtained from the corresponding author.

## Abbreviations

AOR: Adjusted odds ratio
BDHS: Bangladesh Demographic and Health Survey
CI: Confidence interval
COR: Crude odds ratio
RH: Reproductive health
SDGs: Sustainable Development Goals
SRH: Sexual and reproductive health

## References

[1] United Nations International Children’s Emergency Fund (UNICEF) (2016). Adolescent health.

[2] Crichton J, Ibisomi L, Gyimah SO (2012). Mother-daughter communication about sexual maturation, abstinence and unintended pregnancy: experiences from an informal settlement in Nairobi, Kenya. J Adolesc.

[3] Looze M, Constantine N, Jerman P, Vermeulen-Smit E, Bogt T (2015). Parent-adolescent Sexual Communication and its association with adolescent Sexual behaviors: a nationally representative analysis in the Netherlands. J Sex Res.

[4] Yousri Y, Mamdouh HM, Nahla AT, Sally S, EIN N (2013). Mother–daughter communication about sexual and reproductive health in Alexandria, Egypt. Breaking the silence: learning about youth Sexual and Reproductive Health in Egypt.

[5] Jejeebhoy SJ, Santhya KG (2011). Parent-child communication on sexual and reproductive health matters: perspectives of mothers and fathers of youth in India.

[6] King BM, Lorusso J (1997). Discussions in the home about sex: different recollections by parents and children. J Sex Marital Ther.

[7] Pluhar EI, Dilorio CK, McCarty F (2008). Correlates of sexuality communication among mothers and 6-12-year-old children. Child Care Health Dev.

[8] Ayalew M, Mengistie B, Semahegn A (2014). Adolescent-parent communication on sexual and reproductive health issues among high school students in Dire Dawa, eastern Ethiopia: a cross sectional study. Reprod Health.

[9] United Nations International Children’s Emergency Fund (UNICEF) (2015). Analysis of the situation of children and women in Bangladesh 2015.

[10] Huda FA, Chowdhuri S, Robertson Y, Islam N, Sarker BK, Azmi AJ, Reichenbach L (2013). Understanding unintended pregnancy in Bangladesh: Country profile report, STEP UP research report.

[11] Huda FA, Chowdhuri S, Sarker BK, Islam N, Ahmed A (2014). Prevalence of unintended pregnancy and needs for family planning among married adolescent girls living in urban slums of Dhaka, Bangladesh, STEP UP research report.

[12] World Health Organization (WHO) (2011). Strategic direction for improving Adolescent Health in South-East Asia Region.

[13] National Institute of Population Research and Training (NIPORT), Mitra and Associates, & ICF International (2016). Bangladesh Demographic and Health Survey 2014.

[14] Ministry of Health and Family Welfare, Bangladesh. Success factors for women's and children's health. 2015. https://www.who.int/pmnch/knowledge/publications/bangladesh.pdf.

[15] United Nations International Children’s Emergency Fund (UNICEF) (2007). Maternal health in Bangladesh.

[16] Jerman P, Constantine N (2010). Demographic and psychological predictors of parent-adolescent communication about sex: a representative statewide analysis. J Youth Adolesc.

[17] DiIorio C, Resnicow K, Dudley W, Thomas S, Wang DT, Van Marter DF, Manteuffel B, Lipana J (2000). Social cognitive factors associated with mother-adolescent communication about sex. J Health Commun.

[18] Schouten BC, Putte B, Pasmans M, Meeuwesenn L (2007). Parent-adolescent communication about sexuality: the role of adolescents, beliefs, subjective norm and perceived behavioral control. Patient Educ Couns.

[19] Wikipedia. Chittagong; https://en.wikipedia.org/wiki/Chittagong. Accessed 12 Dec 2017.

[20] Tolossa D, Medhin G, Legesse M (2014). Community knowledge, attitude, and practices towards tuberculosis in Shinile town, Somali regional state, eastern Ethiopia: a cross-sectional study. BMC Public Health.

[21] Feldman SS, Rosenthal DA (2000). The effect of Communication characteristics on family members’ perceptions of parents as sex educators. J Res Adolesc.

[22] Shiferaw K, Getahun F, Asres G (2014). Assessment of adolescents’ communication on sexual and reproductive health matters with parents and associated factors among secondary and preparatory schools’ students in Debremarkos town, North West Ethiopia. Reprod Health.

[23] Kim YM, Kols A, Nyakauru R, Marangwande C, Chibatamoto P (2001). Promoting Sexual responsibility among young people in Zimbabwe. Int Fam Plan Perspect.

[24] Eisenberg ME, Sieving RE, Bearinger LH, Swain C, Resnick MD (2006). Parents’ communication with adolescents about sexual behavior: a missed opportunity for prevention?. J Youth Adolesc.

[25] Dilorio C, Kelley M, Hockenberry-Eaton M (1999). Communication about sexual issues: mothers, fathers, and friends. J Adolesc Health.

[26] Fanta M, Lemma S, Sagaro GG, Meskele M (2016). Factors associated with adolescent-parent communication regarding reproductive health issues, among high school and preparatory students in Boditi town, southern Ethiopia: a cross-sectional study. Patient Intelligence.

[27] Manu A, Mba CJ, Asare GQ, Odoi-Agyarko K, Asants RKO (2015). Parent-child communication about sexual and reproductive health: evidence from the BrongAhafo region, Ghana. Reprod Health.

[28] Dessie Y, Berhane Y, Worku A (2015). Parent-adolescent Sexual and Reproductive Health Communication is very limited and associated with adolescent poor behavioral beliefs and subjective norms: evidence from a community based cross-sectional study in eastern Ethiopia. PLoS One.

[29] Melaku YA, Berhane Y, Kinsman J, Reda HL (2014). Sexual and reproductive health communication and awareness of contraceptive methods among secondary school female students, northern Ethiopia: a cross-sectional study. BMC Public Health.

[30] Jordan TR, Price J, Fitzgerald S (2000). Rural parents’ Communication with their teen-agers about Sexual issues. J Sch Health.

[31] Stidham-Hall K, Moreau C, Trussell J (2012). Patterns and correlates of parental and formal Sexual and Reproductive Health Communication for adolescent women in the United States, 2002-2008. J Adolesc Health.

[32] Wamoyi J, Wight D, Plummer M, Mshana GH, Ross D (2010). Transactional sex amongst young people in rural northern Tanzania: an ethnography of young women's motivations and negotiation. Reprod Health.

[33] Directorate General of Health Services (DGHS) (2016). Health Bulletin 2016.

[34] Rogers EM (1973). Communication strategies for family planning.

[35] Lefkowitz E (2002). Beyond the yes-no question: measuring parent-adolescent Communication about sex. New Dir Child Adolesc Dev.

